# Probenecid is a dual-acting antiviral/anti-inflammatory therapy for diverse respiratory viruses

**DOI:** 10.3389/fphar.2026.1765763

**Published:** 2026-04-29

**Authors:** Ralph A. Tripp, Les P. Jones, David E. Martin

**Affiliations:** 1 Department of Infectious Diseases, University of Georgia, Athens, GA, United States; 2 TrippBio, Jacksonville, FL, United States

**Keywords:** anti-inflammatory, anti-viral, direct-acting, host-directed, inflammation, MAPK signaling, probenecid

## Abstract

Probenecid is a host-directed drug that inhibits virus replication. Studies exploring how probenecid affects viral replication have demonstrated that it inhibits mitogen-activated protein kinase (MAPK) signaling pathways, which are important for the virus’s replication and inflammation, and disrupt the formation of the activator protein-1 (AP-1) transcription complex, which regulates gene expression and inflammation. The modulation of MAPK signaling and suppression of the inflammasome response yields a dual anti-inflammatory and antiviral effect. The anti-inflammatory effects of probenecid, achieved through modulation of MAPK signaling and suppression of inflammasome activation, can decrease viral replication and reduces an excessive immune response. This dual mechanism could be advantageous for treating viruses where hyperinflammation plays a significant role in severe disease symptoms. This review suggests that probenecid’s anti-inflammatory activity, mediated by inhibition of MAPK pathways, suppression of NLRP3 inflammasome activation, and reduction of proinflammatory cytokines (such as IL-6, TNF-α, and IL-1β), may help manage viral infections by limiting immune-mediated tissue damage and enhancing clinical outcomes, in addition to its antiviral effects.

## Overview of the proinflammatory response to infection

The proinflammatory response to infectious diseases is a host defense mechanism that involves the release of inflammatory mediators, such as cytokines and chemokines, to coordinate immune cell activity against pathogens (Medzhitov, 2021; Chen et al., 2017). Proinflammatory cytokines, such as IL-1β and IL-6, are key in initiating the immune response to infectious agents (Dinarello, 2009; Tanaka et al., 2014). They promote the recruitment and activation of immune cells, increase vascular permeability, and induce fever, all of which are essential for fighting off invading pathogens (Medzhitov, 2021; Netea et al., 2000).

IL-1β is produced during infection, tissue damage, or in response to other inflammatory stimuli (Medzhitov, 2021; Ford et al., 2025). IL-1β facilitates antibacterial and antiviral effects. IL-1β and the P2X7 receptors are connected in immune signaling (Giuliani et al., 2017). P2X7 receptor activation can trigger NLRP3 inflammasome activation, which then cleaves and releases the active form of IL-1β through various cellular mechanisms (Bockstiegel et al., 2023). This relationship makes the P2X7 receptor an important target for controlling inflammation. IL-1β requires inflammasome activation and caspase-1 cleavage following NF-κB nuclear translocation, which promotes the expression of pro-IL-β and NLRP3-associated proteins (Zhang et al., 2020). This activation step leads to NLRP3 inflammasome assembly and the conversion of pro-caspase-1 to caspase-1, which processes pro-IL-1β into its active form, IL-1β. IL-6 has diverse effects on inflammation, the immune response, and blood cell formation. Macrophages secrete IL-6 in response to pathogen-associated molecular patterns (PAMPs). PAMPs bind to pattern recognition receptors (PRRs), including Toll-like receptors (TLRs). Downstream of these receptors, MAPKs help drive transcription of inflammatory cytokines.

Reducing proinflammatory responses driven by IL-1β and IL-6 to improve patient outcomes is challenging. Multiple studies support IL-6 as a key biomarker of disease progression (Xie et al., 2025). It has been shown that higher IL-6 levels are significantly linked to increased risk of diseases such as adverse cardiovascular events (Mehta et al., 2024). This aligns with earlier research that identifies IL-6 as a central component of the inflammatory response. Anti-inflammatory treatments targeting IL-6 shows promise in decreasing inflammation and improving disease outcomes. Future studies should clarify the context-specific dynamics of IL-6 and IL-1β signaling and identify patient groups most likely to benefit from targeted cytokine inhibition. Clinical trials evaluating the effectiveness of IL-6 and IL-1β inhibitors across diverse patient groups are crucial for confirming their therapeutic potential and determining the most effective treatment approaches.

## The antiviral activity of probenecid

RNA interference (RNAi) is a post-transcriptional gene-silencing mechanism that uses double-stranded RNA to direct homology-dependent suppression of gene expression by degrading the gene’s messenger RNA (mRNA). The pairing of RNAi with genomic sequencing has enabled the construction of genome-scale RNAi libraries. RNAi enables systematic high-throughput genome-wide cell-based analysis. RNAi has been successfully used to probe the virus-host interface and to understand the requirements for host gene expression necessary for virus replication (Panda and Cherry, 2012; Tripp and Mark Tompkins, 2015). Results from these screens have led to the discovery of druggable and key host genes.

RNAi screens and validation in human A549 respiratory epithelial cells infected with influenza virus strains have identified host genes required for influenza replication. One host gene identified was the Organic Anion Transporter-3 gene (OAT3), a member of the solute carrier (SLC)22 family (Perwitasari et al., 2013). Transfection of A549 cells with small interfering RNAs (siRNAs) targeting OAT3 silenced influenza replication with an IC50 ∼10 nM. As OAT3 is necessary for influenza replication, a drug that inhibits OAT3, was investigated for its antiviral properties. Studies showed that probenecid, a classical clinical inhibitor of OAT3, reduced OAT3 mRNA and protein levels, and that probenecid treatment, *in vitro* and *in vivo*, reduced influenza lung titers with IC50s in the picomolar-nanomolar range, suggesting a mechanism that extends beyond just OAT3 inhibition. Further studies with a range of viruses have shown that probenecid has a unique, host-directed antiviral mechanism of action with broad-spectrum efficacy. For example, probenecid treatment inhibited the replication of several important respiratory viruses, including SARS-CoV-2, influenza H5 and H7 strains, human metapneumovirus (HMPV), and Respiratory Syncytial Virus (RSV) (Murray et al., 2022; Bergeron et al., 2024; Murray et al., 2024). This broad antiviral activity is linked to mitogen-activated protein kinase (MAPK) signaling cascades that regulate a wide range of cellular processes. MAPK pathways, when activated by viral infection, initiate a three-tiered kinase cascade leading to a wide range of cellular responses. Viruses and inflammatory signals activate the MAPK family, which includes p38 kinase, c-Jun N-terminal kinase (JNK), and extracellular signal-regulated kinase (ERK) (Chen et al., 2021; Jones et al., 2025b). JNK and p38 have overlapping roles but distinct functions. For example, p38 is involved in pro-inflammatory responses, where its activation leads to the phosphorylation of numerous transcription factors that regulate pro-inflammatory mediators, such as TNF-α and RANTES (Zarubin and Han, 2005). ERK primarily regulates cell proliferation and differentiation, whereas JNK activation is critical for apoptosis (Yang et al., 2014). During viral infection, viral and inflammatory signaling pathways converge with MAPKs to regulate gene expression and promote cytokine production. Viruses hijack MAPK pathways to aid in their replication and pathogenesis. Every virus activates MAPK pathways in distinct ways. For example, the influenza A virus activates the ERK pathway for viral replication, while other viruses, such as RSV, activate the p38 MAPK pathway (Yu et al., 2020). Probenecid can inhibit the phosphorylation of JNK and downstream phosphorylation of the canonical JNK substrate, c-jun, a critical component of the activator protein-1 (AP-1) transcription complex needed for virus replication in A549 cells (Jones et al., 2024). The inhibition of JNK activity by probenecid is associated with the accumulation of transcription factor hepatocyte nuclear factor-4 (HNF-4). HNF4 is also regulated post-transcriptionally by extracellular signal-regulated kinase (ERK) (Jones et al., 2024). Importantly, HNF4 has been shown to regulate OAT3 expression (Ogasawara et al., 2007), and OAT3 is necessary for influenza replication (Perwitasari et al., 2013). MAPK and Inflammasome Signaling in Viral Infection and the Dual Antiviral/Anti-Inflammatory Actions of Probenecid.

**FIGURE 1:**
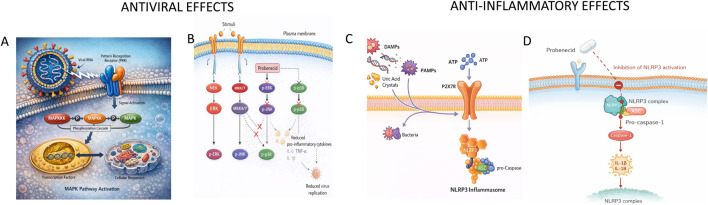
**(A)** Illustration of a virus (e.g., RSV) interacting at the plasma membrane with PRR and inducing MAPKs. **(B)** Under the plasma membrane, there are the 3-tiered MAPK cascades (ERK, JNK, p38), terminating with phospho-activated ERK, JNK, and p38, and showing where probenecid inhibits at the ERK/JNK activation step, resulting in reduced virus replication and reduced cytokine production (IL-6, TNF-α). **(C)** Inflammatory signals (DAMPs/PAMPS) interact with plasma membrane receptors, tickling P2X7R with ATP to activate the NLRP3 inflammasome. **(D)** NLRP3 inflammasome complex (NLRP3/ASC/Cas-1) activation resulting in secretion of IL-1β/IL-18 and inducing pyroptosis, and how probenecid affects this step to reduce IL-1β maturation/secretion and inflammation.

As illustrated in Figure 1, the antiviral activity of probenecid is mechanistically linked to modulation of mitogen-activated protein kinase (MAPK) cascades and downstream inflammasome signaling that coordinate viral replication and inflammatory amplification. MAPK pathways are activated by viral and inflammatory stimuli and propagate signals via a three-tiered kinase cascade to regulate transcription, proliferation, stress responses, and cytokine production (Chen et al., 2021; Jones et al., 2025b; Keshet and Seger, 2010). The principal branches - ERK1/2, JNK1/2, and p38 - have distinct but overlapping functions. p38 primarily drives pro-inflammatory transcription by phosphorylating factors that regulate mediators such as TNF-α and RANTES (Zarubin and Han, 2005). ERK is classically associated with proliferation and differentiation, whereas JNK mediates stress signaling and apoptosis (Chuang et al., 2000; Yang et al., 2014). Extensive crosstalk exists: JNK can enhance ERK-dependent proliferation, while ERK can counteract JNK-driven apoptosis. ERK signaling may be either pro- or anti-inflammatory depending on cellular context and has been shown to suppress NLRP3 activation under specific conditions (Chen et al., 2021).

During infection, viral and host-derived inflammatory signals converge on MAPKs to regulate gene expression and cytokine production. Viruses exploit these pathways in a pathogen-specific manner. Influenza A virus requires ERK activation for replication, whereas respiratory syncytial virus (RSV) preferentially activates p38 MAPK (Yu et al., 2020). Thus, MAPKs serve as central nodes linking viral sensing to replication and inflammatory output. Probenecid interferes with this network by inhibiting MAPK phosphorylation. It suppresses JNK activation and downstream phosphorylation of c-Jun, a core component of the AP-1 complex required for viral replication in A549 cells (Jones et al., 2024). JNK inhibition is associated with the accumulation of hepatocyte nuclear factor-4 (HNF-4), which is post-transcriptionally regulated by ERK (Jones et al., 2024). HNF-4 controls expression of organic anion transporter 3 (OAT3) (Ogasawara et al., 2007), a host factor required for influenza replication (Perwitasari et al., 2013). Probenecid also inhibits ERK phosphorylation during viral infection, including RSV (Jones et al., 2024), thereby disrupting MAPK-dependent transcriptional programs that support viral replication.

MAPK signaling intersects with purinergic and inflammasome pathways that amplify inflammation. Pannexin-1 (PANX1) regulates extracellular ATP release, promoting inflammatory signaling (Jones et al., 2025a). ATP activates the P2X7 receptor (P2X7R), which, together with NF-κB and MAPK-dependent priming, drives activation of the NLRP3 inflammasome (Di Virgilio et al., 2017). PANX1 activity enhances secretion of proinflammatory cytokines such as IL-1β and IL-6 (Bravo et al., 2025). NLRP3 inflammasome, composed of NLRP3, ASC, and caspase-1 (Benetti et al., 2013), senses pathogen- and damage-associated stress and mediates maturation of IL-1β and IL-18 (Bulte et al., 2023). While essential for host defense, excessive NLRP3 activation contributes to pathological inflammation in metabolic disease, gout, and severe viral infection (Wang et al., 2025). Probenecid attenuates this inflammatory axis at multiple levels. It modulates PANX1 expression, limiting extracellular ATP release and downstream P2X7R activation (Jones et al., 2025a). By suppressing P2X7R-dependent signaling, probenecid inhibits NLRP3 assembly and caspase-1 activation, reducing IL-1β production and hyperinflammation during severe influenza infection (Jones et al., 2025a). Notably, probenecid also reduces inflammation and enhances bacterial clearance in a mouse model of *Pseudomonas aeruginosa* pneumonia without direct antibacterial effects, indicating that its anti-inflammatory activity can be mechanistically distinct from its antiviral effects (Wonnenberg et al., 2014). Collectively, MAPK cascades and the PANX1–P2X7R–NLRP3 axis form an integrated network governing viral replication and inflammatory amplification. By inhibiting ERK and JNK phosphorylation, modulating HNF-4/OAT3 signaling, and suppressing ATP-driven inflammasome activation, probenecid exerts coordinated antiviral and anti-inflammatory effects.

## Proinflammatory cytokines inhibited by probenecid

Probenecid suppresses proinflammatory cytokine production by convergently inhibiting MAPK signaling (ERK and JNK), PANX1-mediated ATP release, and NLRP3 inflammasome activation (Jones et al., 2025a). Through these mechanisms, probenecid reduces the production and release of key inflammatory cytokines, including IL-1β, IL-6, and TNF-α. Inhibition of ERK and JNK phosphorylation reduces MAPK-dependent transcription of inflammatory mediators, indirectly lowering IL-6 and TNF-α levels. Probenecid also specifically inhibits caspase-1 activation by preventing caspase-1 auto-cleavage following inflammasome assembly, thereby blocking maturation of IL-1β. Importantly, probenecid does not reduce pro-IL-1β protein expression, indicating that its primary effect is on cytokine processing and release rather than transcription. Some studies suggest that probenecid does not directly inhibit TNF-α production but instead reduces TNF-α levels indirectly by modulating upstream MAPK signaling (Jones et al., 2025a).

Inflammatory amplification is further driven by damage-associated molecular patterns (DAMPs), which are released either passively during cell death or actively from stressed but viable cells (Lin et al., 2025). Several forms of cell death, including necrosis, apoptosis, ferroptosis, and pyroptosis, contribute to DAMP release. Pyroptosis, a caspase-dependent inflammatory cell death pathway, is particularly relevant in viral infection. During pyroptosis, NLRP3 activation leads to caspase-1–mediated cleavage of gasdermin proteins, whose N-terminal fragments form membrane pores that allow the release of IL-1β and IL-18 (Kesavardhana et al., 2020; Liu et al., 2024). By inhibiting PANX1 and limiting extracellular ATP signaling, probenecid reduces inflammasome priming and dampens pyroptotic pore formation, thereby further restricting cytokine release and inflammatory tissue damage.

## Probenecid modulation of inflammatory pathways to different infectious diseases

Probenecid modulates inflammatory pathways by inhibiting inflammasome proteins, such as NLRP1 and NLRP3, and blocking the MAPK signaling pathway (Jones et al., 2025a). Probenecid treatment reduces pro-inflammatory cytokines, oxidative stress, and pyroptosis, and can also inhibit PANX1, which promotes inflammation (Jones et al., 2025a; Wang et al., 2025). This suggests that probenecid could be a potential therapy for various inflammatory conditions by targeting multiple pathways. The combined effects of probenecid demonstrate significant anti-inflammatory properties and highlight its ability to modulate multiple inflammatory pathways, suggesting potential uses in various inflammatory diseases. These anti-inflammatory mechanisms have been demonstrated across several infectious models. In viral infection models using Vero E6 cells (isolated from the kidney of an African green monkey), human respiratory cell lines (A549 respiratory epithelial cells), and normal human bronchoepithelial (NHBE) cells, probenecid treatment inhibits JNK and ERK phosphorylation, thereby reducing IL-1β levels. IL-1β reduction during viral infection also depends on inflammasome inhibition, not only on MAPK inhibition, which modulates cytokine expression. Clinical COVID-19 data indicate a correlation between probenecid treatment and reductions in systemic inflammation markers (like temperature) and COVID-19 symptoms (Martin et al., 2023). In a *Pseudomonas* pneumonia infection model, probenecid reduced lung cytokine responses by limiting inflammasome activation and IL-1β secretion, without any antimicrobial activity, thereby improving outcomes (Wonnenberg et al., 2014). These findings suggest that probenecid could be a promising therapy for infectious diseases marked by hyperinflammatory responses (such as viral pneumonia or bacterial sepsis).

## Clinical translation of the antiviral and anti-inflammatory effects

Probenecid has been used clinically since the early 1950s, originally as a uricosuric agent for the treatment of gout and later as an adjunct therapy to prolong penicillin (and eventually β-lactams) antibiotic exposure by inhibiting renal tubular organic anion transport (Beyer et al., 1950; Boger et al., 1955; Robbins et al., 2012). Patients with gout can receive doses of probenecid up to 2000 mg/day and be treated for months or years. Despite the long-term use and relatively high dose burden, probenecid is generally regarded as having a benign safety profile. Common adverse effects are usually mild and reversible and include headache, nausea, vomiting, loss of appetite, and other gastrointestinal discomforts. Cutaneous rash or hypersensitivity reactions are reported in a small proportion of patients (Probenecid Package Insert). Significant hemopoietic, renal, or hepatic toxicity appears uncommon, although rare serious hypersensitivity reactions (including severe rash) have been described, so standard clinical monitoring and patient counseling are recommended. Because probenecid alters renal tubular handling of organic anions (the basis for its use as a pharmacokinetic enhancer for penicillin and certain β-lactam antibiotics), it has numerous, well–characterized drug–drug interactions, which are manageable through dose adjustment, spacing of administration, or selection of alternative agents.

Probenecid administration, as a host-directed antiviral, has been limited to a single report in a randomized Phase 2 study in non-hospitalized adults with mild–to-moderate COVID-19, where twice-daily doses of 500 or 1000 mg for 5 days produced a dose-dependent reduction in viral RNA and time to symptom resolution (Martin et al., 2023). There was a low incidence of treatment-emergent adverse events (∼12%), all mild, with no serious events or discontinuations reported (Martin et al., 2023), consistent with the long-established clinical history of probenecid. Reported antiviral IC50 values for probenecid vary across viruses and experimental systems, spanning picomolar to low-micromolar ranges for respiratory viruses (Tripp and Martin, 2015), whereas data from the *Pseudomonas aeruginosa* pneumonia model (where probenecid has no known inhibitory effect) indicate that anti-inflammatory benefits (reduced IL-1β and improved bacterial clearance) can occur at exposures that do not require direct antimicrobial activity, suggesting that clinically relevant anti-inflammatory effects may be achieved at, or even below, concentrations needed for maximal antiviral activity. Together, these data support the feasibility of translating probenecid’s dual antiviral/anti-inflammatory activity into outpatient treatment strategies that leverage existing dosing paradigms, with attention to standard drug-interaction management and patient selection based on concomitant medications and comorbidities.

## Future directions

More studies are needed to identify which features contribute to dysregulated proinflammatory cytokine pathways and to optimize the timing and dosing of probenecid. Combining probenecid with drugs that modulate inflammation may be the next step in treatment. This is supported by probenecid’s ability to reduce key cytokines (IL-1β, IL-18, IL-6, TNF-α) by inhibiting MAPK and the inflammasome, offering promise for treating infection-driven hyperinflammation.
